# Geographic and Socioeconomic Variability in Commercially Negotiated Pricing for Hip and Knee Arthroplasty

**DOI:** 10.2106/JBJS.OA.25.00333

**Published:** 2026-01-26

**Authors:** Jonathan Wang, Andrew B. Harris, Wenyu Yang, Yang Wang, Savyasachi C. Thakkar, Amit Jain

**Affiliations:** 1 School of Medicine, Johns Hopkins University, Baltimore, Maryland; 2 Department of Orthopedic Surgery, Johns Hopkins University, Baltimore, Maryland; 3 Department of Biomedical Engineering, Johns Hopkins University, Baltimore, Maryland; 4 Bloomberg School of Public Health, Johns Hopkins University, Baltimore, Maryland; 5 Carey School of Business, Johns Hopkins University, Baltimore, Maryland

## Abstract

**Background:**

Total hip and knee arthroplasty (THA/TKA) are among the most common and costly orthopedic procedures in the United States. While hospital-level pricing drivers are known, the influence of community socioeconomic and geographic factors remains unclear. This study aimed to evaluate both hospital-level and community-level predictors of THA/TKA pricing.

**Methods:**

A retrospective cross-sectional analysis used commercially negotiated THA/TKA inpatient hospital prices (Medicare Severity-diagnosis-related group 470) from 2,286 US hospitals encompassing 5,946 prices across 5 major commercial payers (Turquoise Health, March 2024). Prices are defined as claims costs reimbursed by payers, excluding surgeon, anesthesia, and outpatient fees. Hospital price data were linked to county-level socioeconomic, clinical need (e.g., arthritis prevalence), and hospital structural characteristics (e.g., bed count). Multivariate linear regression assessed associations between hospital/community factors and log-transformed prices.

**Results:**

Negotiated THA/TKA prices were significantly higher in rural than urban counties. Multivariate analysis found that higher prices were associated with larger hospital bed count (+5.20% per standard deviation [SD], p < 0.001), higher Case Mix Index (+5.34% per SD, p = 0.002), and counties with minority population populations (+3.19% per SD, p = 0.001) and uninsured rates (+2.91% per SD, p = 0.019). By contrast, higher arthritis prevalence (−3.13% per SD, p < 0.001) and Social Deprivation Index scores (−7.23% per SD, p < 0.001) were associated with lower prices, which suggests that high-need populations face lower hospital reimbursement or reflect price adjustments in response to local economic capacity.

**Conclusions:**

Commercially negotiated THA/TKA prices are influenced by both hospital structural factors and the demographics of the surrounding community. Higher prices in areas with larger minority populations and more uninsured residents may reflect systemic drivers of geographic and demographic disparities in healthcare pricing.

**Level of Evidence:**

Level III. See Instructions for Authors for a complete description of levels of evidence

## Introduction

Total hip and knee arthroplasty (THA/TKA) are major orthopedic procedures, crucial for restoring mobility and quality of life^1,2^. Simultaneously, they rank among the most cost-intensive surgeries in the United States, with over 2 million procedures projected annually by 2040^3^. Commercial pricing remains highly variable and opaque, with negotiated rates differing widely across hospitals and payers^4-7^. While prior research has demonstrated the influence of hospital-level factors, the role of community-level socioeconomic and geographic characteristics is less understood^8-11^.

Socioeconomic vulnerability (e.g., income levels, insurance coverage) is poised to significantly impact prices and, by extension, patient access^12-14^. Limited affordability of THA/TKA can delay care, worsen mobility and quality-of-life outcomes, and exacerbate existing health disparities, particularly among medically underserved populations^15-17^. Historically, the lack of price transparency has hindered exploration of how social, economic, and clinical context shapes the cost of essential surgical care^18-20^.

The 2022 Centers for Medicare & Medicaid Services (CMS) Transparency in Coverage (TiC) Rule now requires insurers to publicly disclose commercially negotiated prices through machine-readable files, offering a unique opportunity to examine such intersections^21^. This study aims to independently characterize hospital-level and community-level predictors of THA/TKA price variation. By linking negotiated pricing patterns to demographics and hospital characteristics, such analysis provides actionable insights for orthopedic practitioners, policymakers, and payers to improve price transparency, promote value-based care, and ensure equitable access to essential surgical procedures.

## Methods

### Health Claims Pricing Data

A retrospective cross-sectional analysis used national commercially negotiated inpatient hospital claims for THA/TKA grouped under Medicare Severity-diagnosis-related group (MS-DRG) 470. Data were sourced from Turquoise Health, which aggregates insures’ publicly available machine-readable files submitted under the CMS TiC Rule^22^. Claims were current as of March 2024 and included reimbursement amounts from 5 major commercial payers: Aetna, Anthem, Blue Cross Blue Shield (BCBS), Cigna, and UnitedHealthcare. Hereafter, “prices” refer to these commercially negotiated inpatient hospital claims costs, excluding surgeon, anesthesia, and outpatient fees and represent the full contracted amount reimbursed by payers, including both insurer and patient contributions.

MS-DRG 470 was selected because Current Procedural Terminology-based pricing in transparency files is often incomplete or inconsistently coded^23,24^. For hospital-payer pairs with multiple reported prices, the median value was used. Extreme outliers (top and bottom 1%) were excluded to reduce the influence of reporting artifacts^25,26^. The cutoff was determined heuristically and applied equally to both extremes of the distribution to reduce the impact of highly unrepresentative values. Such outliers may stem from data entry mistakes, atypical contractual agreements with payers, or institution-specific pricing practices that do not reflect broader market norms.

### Hospital Characteristic Data

Hospital-level characteristics were derived from the CMS Hospital General Information file and supplemented with additional administrative data sets to capture structural, operational, and system-level attributes relevant to pricing practices. Hospital size was quantified using total bed count and clinical complexity by the Case Mix Index (CMI)^27,28^. The CMI represents the average DRG weight for a hospital's inpatient Medicare claims, providing a standardized proxy for clinical severity and resource intensity: higher values indicate more complex case profiles.

Ownership was categorized as nonprofit, for-profit, or government, and teaching status was determined by Council of Teaching Hospitals membership. System affiliation and size were extracted from the Agency for Healthcare Research and Quality Comparative Health System Performance Initiative database^29^. Hospitals affiliated with a multihospital system were identified, and the size of each health system was operationalized as the total number of acute care hospitals within the network. Systems were stratified into 5 ordinal categories based on size: Solo (1), Small (2-5), Medium (6-15), Large (16-40), and Very Large (41+). Cutoffs were based on literature-informed thresholds distinguishing single hospitals, regional networks, and large multistate systems^30,31^.

### Community-Health and Socioeconomic Data

Geographic and socioeconomic data were integrated from multiple sources, including the Centers for Disease Control and Prevention (CDC) Population Level Analysis & Community Estimates database, the Agency for Healthcare Research and Quality Social Determinants of Health database, and the Robert Graham Center Social Deprivation Index (SDI)^32-36^. The SDI combines measures of poverty, education, single-parent households, rented or overcrowded housing, vehicle access, and unemployment^37^. Hospitals were geographically linked at the ZIP Code Tabulation Area level to characterize the surrounding community environment. Area-level covariates included the SDI score (range 0-100), uninsured rate, and minority population share. Community-level clinical burden was assessed using adult prevalence estimates of arthritis disability, as reported in the CDC Population Level Analysis & Community Estimates database. These variables were selected to capture the socioeconomic disadvantage and health status of the communities served by each hospital.

### Multivariate Linear Regression Modeling

Multivariate linear regression evaluated associations between hospital-level and community-level factors with prices at the hospital-insurer level. Prices were log-transformed to address right-skewed distribution. Independent variables were selected for theoretical relevance and univariable screening (p < 0.20). Hospital-level predictors included ownership status, teaching designation, system affiliation, bed count, and CMI, while community-level predictors including minority population percentage, arthritis prevalence, uninsured rate, and SDI.

Fixed effects for geographic region (Northeast, Midwest, South, West) captured regional variation, and insurer fixed effects controlled for payer-specific pricing differences. Standardized beta coefficients (β) were reported with 2-sided p < 0.05 indicating statistical significance, and multiple comparison adjustments applied where appropriate. Sensitivity analyses sequentially excluded individual community-level covariates (Minority Population %, SDI, Arthritis Prevalence %, and Uninsured Rate %) and evaluated an alternative log_10_ price transformation to assess robustness.

## Results

### Hospital and Community-Level Predictors of Pricing Variation

#### Model Diagnostics and Overall Performance

Multivariate modeling was conducted on 5,946 THA/TKA prices across 2,286 hospitals (Table I). Adjusted R^2^ was 0.284. Residual diagnostics confirmed approximate linearity and no major violations of ordinary least squares assumptions. Complete regression results are provided in Appendix A.

**TABLE I:** Descriptive Characteristics of the 2,286 Hospitals and 2,096 Zip-Codes Included in the Multivariate OLS Regression

Measure	Subcategory	N (%)	Mean ± SD	Mean Price ± SD (USD)
Hospital level characteristics				
Hospital system size	Solo (1 hospital)	370 (16.2%)		22,276.85 ± 10,520.03
	Small (2-5 hospitals)	297 (13.0%)		29,286.89 ± 11,524.00
	Medium (6-15 hospitals)	524 (22.9%)		31,634.97 ± 11,545.07
	Large (16-40 hospitals)	413 (18.1%)		32,470.21 ± 16,131.03
	Very large (41+ hospitals)	682 (29.8%)		30,265.11 ± 11,333.96
Total bed count			263.7 ± 252.9	
Teaching hospital	No	1,393 (60.9%)		28,154.83 ± 12,833.08
	Yes	893 (39.1%)		31,745.45 ± 12,257.71
Hospital ownership	Government	128 (5.6%)		26,214.89 ± 12,570.38
	Non-profit	1,566 (68.5%)		30,546.46 ± 12,837.44
	Other	592 (25.9%)		27,664.04 ± 12,148.38
Case Mix Index (CMI)			1.83 ± 0.42	
Insurer	Aetna	1,123 (18.9%)		31,594.20 ± 14,410.12
	Anthem	524 (8.8%)		32,307.97 ± 12,275.65
	Blue cross blue shield	1,207 (20.3%)		31,104.42 ± 14,222.82
	Cigna	1,315 (22.1%)		31,566.25 ± 15,600.24
	United	1,777 (29.9%)		28,269.38 ± 14,370.82
Region	Midwest	605 (26.5%)		27,225.93 ± 9,281.37
	Northeast	331 (14.5%)		32,957.46 ± 15,267.35
	South	895 (39.2%)		28,337.06 ± 13,653.83
	West	455 (19.9%)		38,279.63 ± 18,157.2
ZIP-code community characteristics				
Minority population (%)			37.2 ± 23.7	
Arthritis prevalence (%)			27.5 ± 6.0	
Uninsured rate (%)			8.6 ± 5.1	
Social deprivation index score			54.7 ± 28.1	

#### Hospital Characteristics and System-Level Factors

**Fig. 1:**
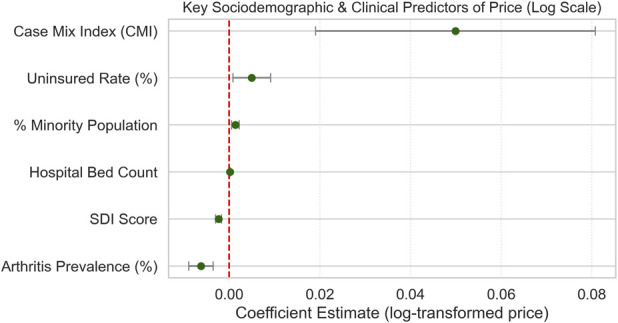
Forest plot of significant continuous predictors of log-transformed THA/TKA prices. The forest plot displays the estimated regression coefficients (β) and their 95% confidence intervals of statistically significant continuous predictors from the multivariate ordinary least squares regression model, assessing the independent association of various hospital- and community-level characteristics with the log-transformed commercially negotiated prices for THA/TKA. The vertical red dashed line at zero indicates no effect. Notably, higher Case Mix Index (CMI), Uninsured Rate (%), Minority Population (%), and Hospital Bed Count are significantly associated with increased THA/TKA prices. Conversely, higher SDI Score and Arthritis Prevalence (%) is significantly associated with lower prices. THA/TKA = Total hip and knee arthroplasty.

**Fig. 2:**
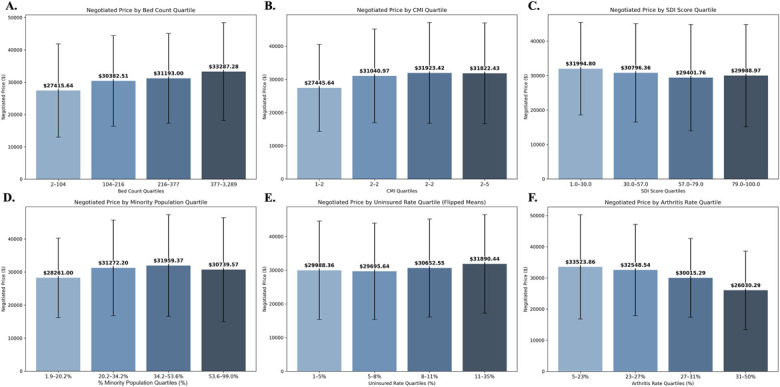
Average commercially negotiated THA/TKA prices by quartile of key sociodemographic and hospital characteristics. A series of bar charts illustrates the average commercially negotiated prices for THA/TKA across quantiles of 6 independent variables: across quantiles of 6 key independent variables: (**Fig. 2-A**) Hospital Bed Count, (**Fig. 2-B**) Case Mix Index (CMI) (**Fig. 2-C**) SDI Score, (**Fig. 2-D**) Minority Population (%), (**Fig. 2-E**) Uninsured Rate (%), and (**Fig. 2-F**) Arthritis Rate (%). For each variable, counties or hospitals were grouped into quartiles based on their distribution, and the average negotiated THA/TKA price within each quantile is displayed. Error bars represent the standard deviation of prices within each quantile. These descriptive analyses visually depict the observed relationships between these characteristics and average THA/TKA prices, complementing the multivariate regression findings by highlighting direct price variations across different levels of these factors. THA/TKA = Total hip and knee arthroplasty.

Teaching designation and ownership were not significant predictors of pricing. Higher bed count (β = 1.95 × 10^−4^, p < 0.001: 95% confidence interval [CI] [1.44 × 10^−4^, 2.41 × 10^−4^]) and clinical complexity (β = 0.052, p = 0.002: 95% CI [0.020-0.084]) were associated with increased price (Figs. 1 and 2-A and 2-B). Compared with single-hospital systems, negotiated prices were higher in multihospital systems: Small (β = 0.192, 95% CI: [0.138-0.246]), Medium (β = 0.315, 95% CI: [0.265-0.362]), Large (β = 0.338, 95% CI: [0.287-0.391]), and Very Large (β = 0.281, 95% CI: [0.234-0.326]), all p < 0.001.

#### Community Socioeconomic and Clinical Context

Community-level characteristics were also consistent predictors of pricing variation (Fig. 1). Hospitals serving areas with higher minority populations (β = 0.00142, p = 0.001: 95% CI [0.000556-0.00228]) or greater uninsured rates (β = 0.00485, p = 0.021: 95% CI [0.000786-0.00904]) had significantly higher prices (Figs. 2-D and 2-E). By contrast, higher arthritis prevalence (β = −0.00610, p ≤ 0.001: 95% CI [−0.00888 to −0.00342]) and increased social deprivation (β = −0.00230, p < 0.001: 95% CI [−0.00297 to −0.00163]) were associated with lower prices (Figs. 2-C and 2-F).

#### Region and Insurer Differences

**Fig. 3:**
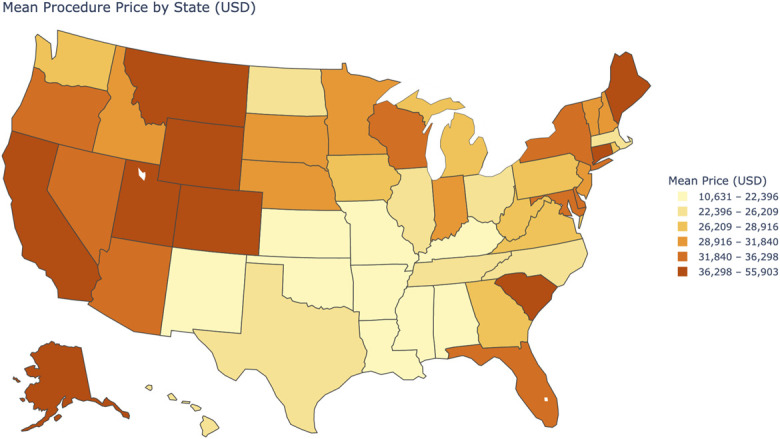
Average commercially negotiated THA/TKA prices by state (USD). This map illustrates the geographic distribution of average commercially negotiated prices for THA/TKA across U.S. states included in the multivariate ordinary least squares regression analysis. Prices are categorized into 6 quantile-based bins, with darker shades indicating higher average prices. This map visually demonstrates the substantial state-level variation in THA/TKA prices, which was accounted for in the regression model through the inclusion of state fixed effects. THA/TKA = Total hip and knee arthroplasty.

Significant variation in negotiated prices was observed across both regions and insurers (Fig. 3). Compared with the South, hospitals in the West (β = 0.292, p < 0.001: 95% CI [0.252-0.333]) and Northeast (β = 0.178, p < 0.001: 95% CI [0.135-0.220]) had significantly higher prices, likely reflecting greater concentration of tertiary centers and higher regional costs. The Midwest showed comparable prices, consistent with more moderate cost structures and lower prevalence of high-cost tertiary referral centers. Using Aetna as the reference, BCBS reported higher prices (β = 0.049, p = 0.004: 95% CI [0.014-0.084]), while UnitedHealthcare demonstrated lower prices (β = −0.149, p < 0.001: 95% CI [−0.184 to −0.114]): Anthem or Cigna did not differ significantly.

#### Sensitivity Analyses

Sensitivity analyses demonstrated that the magnitude, direction, and significance of hospital-level and community-level predictors remained consistent across alternative model specifications. Sequential exclusion of community-level covariates and the use of a log_10_ price transformation yielded qualitatively similar results, confirming the robustness of the primary model (Supplementary Table S1).

### Urban-Rural Price Disparity

Rural counties exhibited significantly higher average negotiated prices ($30,151) compared to their urban counterparts ($21,120), a difference that was statistically significant (p < 0.001). This difference may reflect factors such as lower local competition, higher operational costs, and limited access to high-volume arthroplasty centers in rural settings, potentially contributing to geographic inequities in procedural costs and patient access.

## Discussion

### The Intersection of Market Power and Clinical Complexity

Larger hospitals, reflected by higher bed count and greater system size, were associated with higher negotiated prices, suggesting that resource availability and organizational scale confer market leverage with commercial payers^24,38,39^. Such hospitals may also have flexibility to go out-of-network or selectively contract with insurers, influencing negotiated rates. Furthermore, the association between higher prices and greater clinical complexity indicates that commercial reimbursement is, to some degree, functioning correctly by adjusting for patient care intensity^1^. Notably, traditional descriptors such as teaching status and ownership model were not significant predictors, suggesting that in the modern healthcare marketplace, functional market power and case mix outweigh historical institutional classifications in determining price.

### Socioeconomic Drivers and Cost-Shifting Theory

Community-level characteristics emerged as consistent predictors of negotiated THA/TKA pricing. Of concern is the finding that hospitals serving areas with a higher minority population percentage had significantly higher THA/TKA prices, indicating potential structural inequities within the healthcare marketplace. Chang et al. (2024) similarly found strong positive correlations between hospital-reported commercial prices and community-level social risk factors, including the percentage of Hispanic residents^19^. Potential contributors include historical underinvestment, which limits provider competition, and implicit biases affecting payer-provider negotiations^40,41^.

Higher community rates of uninsurance were also linked to higher prices, consistent with cost-shifting dynamics^42,43^. It further aligns with findings from Chang et al. (2024), who reported that hospitals in communities with higher uninsured rates consistently charged higher prices^19^. Such a dynamic could suggest that communities with high uninsured populations may face a dual burden of reduced access and higher costs for those with private coverage.

### The Paradox of High Need and Lower Reimbursement

Conversely, greater social deprivation and higher arthritis prevalence were associated with lower negotiated prices. This counterintuitive relationship suggests two potential mechanisms. First, hospitals in high-burden, high-need areas may participate more in value-based care or bundled payment programs for THA/TKA, which incentivize and have been linked to lower per-procedure costs^44-48^. Second, pricing in socioeconomically vulnerable communities may reflect implicit market adjustments to local economic capacity^49^. However, lower reimbursement in these settings warrants further study, as it may also signal reduced procedural availability, care quality, or access for the populations that need these services most^50^.

### The Rural-Urban Divide and Geographic Vulnerability

The rural-urban disparity in prices was notable. Rural communities often face physician shortages, limited hospital competition, and higher proportions of elderly or low-income residents, reducing negotiating leverage^8,51,52^. The combination of higher prices and socioeconomic vulnerability may create financial barriers to necessary orthopedic care, delaying treatment and worsening outcomes^53^. These disparities carry important policy implications: while regulatory approaches such as all-payer rate setting may reduce unwarranted price variation, they must account for local context^54,55^.

### Potential Implications

Identifying geographic and community-level pricing drivers offers orthopedic surgeons actionable intelligence to optimize both practice strategy and patient care. High-burden or high-need communities present opportunities to participate in bundled payment or value-based care programs, aligning cost-containment with predictable reimbursement while expanding access for socioeconomically vulnerable patients^56,57^. In addition, recognizing that high prices in minority and uninsured areas may be a structural response to underfunding provides a data-driven basis for advocating for better payer mix management and financial assistance programs to ensure equitable access^58^.

### Limitations

Several limitations warrant consideration. First, this study focuses on negotiated commercial prices for MS-DRG 470, capturing only inpatient reimbursement and excluding outpatient or ambulatory surgical center settings. As THA/TKA procedures increasingly shift to lower-cost environments, this restriction may omit a substantial share of cases, potentially overestimating prices and underrepresenting cross-setting variability.

Although the data set includes 5 major commercial payers, it excludes regional insurers and public programs such as Medicare and Medicaid, limiting applicability in areas with high public coverage. Price variation may also reflect unmeasured institutional or market factors, including physician ownership, hospital–physician integration, payer-specific contracts, and workforce composition. Finally, while TiC data enhance transparency, incomplete compliance and reporting inconsistencies may introduce selection bias^59^.

## Conclusion

The landscape of negotiated THA/TKA pricing in the United States is defined by a complex interaction of hospital market power and community demographics. Higher prices were linked to greater hospital market power, higher minority populations, and higher uninsured rates, while greater arthritis prevalence was associated with lower prices. These findings highlight the need for orthopedic surgeons and healthcare leaders to incorporate community context into pricing and reimbursement strategies to promote value-based care in joint replacement surgery.

## Appendix

Supporting material provided by the authors is posted with the online version of this article as a data supplement at jbjs.org (http://links.lww.com/JBJSOA/B88). This content was not copyedited or verified by JBJS.
